# Evaluation of Cognitive Performance in Patients with Fibromyalgia Syndrome: A Case–Control Study

**DOI:** 10.3390/life14050649

**Published:** 2024-05-20

**Authors:** Francisco G. Fernández-Palacios, Juan C. Pacho-Hernández, César Fernández-de-las-Peñas, Cristina Gómez-Calero, Margarita Cigarán-Méndez

**Affiliations:** 1 Escuela Internacional de Doctorado, Universidad Rey Juan Carlos, 28933 Madrid, Spain; gines.fernandez@urjc.es; 2 Department of Psychology, Universidad Rey Juan Carlos, 28922 Alcorcón, Spain; margarita.cigaran@urjc.es; 3 Department of Physical Therapy, Occupational Therapy, Rehabilitation, and Physical Medicine, Universidad Rey Juan Carlos, 28922 Alcorcón, Spain; cesar.fernandez@urjc.es (C.F.-d.-l.-P.); cristina.gomez@urjc.es (C.G.-C.)

**Keywords:** anxiety, cognitive performance, depression, fibromyalgia syndrome, sleep quality

## Abstract

Patients with fibromyalgia syndrome tend to report deficits in cognitive functions; however, there is no clear consensus on which cognitive domains are impaired. The aim of this study was to compare the differences in cognitive performance between a group of patients with fibromyalgia syndrome and a group of pain-free subjects controlling for the covariables anxiety, depression, and sleep quality. In total, 130 patients with fibromyalgia syndrome and 111 pain-free subjects with an average age of 54.96 years completed the evaluation protocol consisting of sociodemographic data, psychological data, and neurocognitive tests. All data were collected from May 2022 to May 2023. Multivariate analyses of covariance (MANCOVAs) were conducted to assess intergroup differences in all neurocognitive tests. MANCOVA analyses showed that the group of patients with fibromyalgia showed a worse cognitive performance than the group of pain-free subjects after controlling for anxiety, depression, and sleep quality. This study found that fibromyalgia patients exhibited worse cognitive performance and executive function than pain-free subjects. Thus, cognitive performance seems to not be related with anxiety, depression, or sleep quality in our sample of women with FMS.

## 1. Introduction

Fibromyalgia syndrome (FMS) is a complex pain condition affecting up to 6% of the worldwide population [1]. Although its clinical presentation is well described in the literature [2], its etiology is not completely understood. Patients with FMS generally report widespread pain symptoms, fatigue, sleep disorders, emotional disturbances, cognitive dysfunctions, exacerbated pain responses to painful and non-painful stimuli, generalized muscle weakness, decreased physical capacity, and reduced health-related quality of life [3]. The pathogenesis of FMS is complex, and several aspects, including altered nociceptive processing, as well as emotional, psychological, and cognitive features, can interact at the same time [4]. In fact, the presence of central nervous system-associated symptomatology such as depression, anxiety, or sleep disorders can promote chronic pain, and in return, chronic pain can promote these symptoms. For instance, there is evidence supporting a bidirectional relationship between chronic musculoskeletal pain and sleep disorders [5].

Cognitive alterations are self-reported by up to 60% of patients with FMS and can range from overall short- and long-term memory loss (e.g., problem with recalling names or words) to deficit attention and executive function deficits [6]. In fact, this subjective experience of cognitive dysfunction self-perceived by patients with FMS has been called “fibrofog” [7]. Different meta-analyses have reported that cognitive impairments in FMS are heterogeneous and seem to be domain-specific. Wu et al. found large effect sizes for learning/memory and attention/psychomotor speed and medium effect sizes for working memory when comparing women with FMS with healthy women [8]. Furthermore, Bell et al. found moderate-to-large effects for inhibitory control, short-and long-term memory loss, and task switching [9]. Notwithstanding, further studies investigating different multidimensional aspects of cognitive performance are required.

Several factors associated with FMS, i.e., pain, depression, anxiety, catastrophism, hypersomnia, fatigue, and hypervigilance, could affect cognitive performance in patients with FMS, although the results are inconsistent. Some studies reported that depressive or anxiety levels could affect cognitive impairment in patients with FMS [10,11]; however, other authors did not find such an association [12]. Although sleep deprivation has also been found to influence some aspects of cognitive performance, i.e., attention or memory [13,14], the influence of sleep quality on cognitive performance in FMS has been less investigated, and conflicting results have been observed [15,16]. Another pain-related aspect that has already been described in FMS and can also influence cognitive performance is pain hypervigilance [17].

Expanding the understanding of executive changes in FMS controlling by potential pain-related emotional and cognitive factors is relevant for both research and clinical practice with this clinical population. Accordingly, the primary aim of this current study was to compare differences in cognitive performance and executive function between women with FMS and matched pain-free subjects. In contrast to most previous studies, our research approach allowed for a differential analysis of performance with respect to attention, long-term memory, and executive functions of working memory, such as inhibition, processing speed, decision making, and mental planning, controlling for sleep quality, anxiety, and depression. The hypothesis of this study was that the group of FMS patients would report less scores in all neurocognitive tests in comparison to the group of pain-free subjects. Furthermore, it was hypothesized that the cognitive performance of both groups would not be affected by anxiety, depression, or sleep quality.

## 2. Methods

### 2.1. Participants

A consecutive sample of 130 women with FMS diagnosed by their rheumatologist [18] were voluntarily recruited from local announcements at different Fibromyalgia Associations in Madrid (Spain). In addition, a sample of 111 asymptomatic pain-free women without a history of chronic pain disease were recruited via local advertisements in different social networks (Facebook, WhatsApp, and Twitter) and on the bulletin boards of the Faculty of Health Sciences of the Universidad Rey Juan Carlos. The overall inclusion criteria for participation for both groups were as follows: (1) age between 18 and 75 years old; and (2) ability to speak and read Spanish fluently. The exclusion criteria for both groups were as follows: (1) previous whiplash; (2) previous surgery; (3) comorbid underlying medical conditions (e.g., rheumatoid arthritis); (4) neuropathic pain conditions (e.g., radiculopathy, myelopathy); (5) current diagnosis of a psychiatric disorder according to the DSM-V [19] (e.g., major neurocognitive disorders, mild neurocognitive disorders, ASD, ADHD, or schizophrenia that significantly compromises cognitive abilities); and (6) taking medication that can affect cognition, such as antipsychotics, anticonvulsants, and anticholinergics [20].

We permitted the symptomatic use of nonsteroidal anti-inflammatory drugs (NSAIDs) within the FMS group since it is a common medication taken by these patients. Additionally, for the group of pain-free subjects, another exclusion criterion was having a diagnosis of a chronic pain illness.

The sample size was estimated to detect a medium effect using G*Power 3.1.9.7, and it was found that 216 individuals were sufficient for the two groups, with an alpha error of 0.05 and a target power of 0.95, following Cohen’s guidelines for small, medium, and large effects [21]. The sample was composed mostly of Caucasians (97.51%), with secondary education (38.58%), who were married (70.53%), and who were working (43.56%). The average age was 54.96 years (SD = 11.71). The study design was approved by the Ethics Committee of Universidad Rey Juan Carlos (internal record ID: 2508202218222). All participants received information about the study and signed a written informed consent before their inclusion. All data were collected from May 2022 to May 2023 in a single session lasting 90 min in the experimental laboratory of the Universidad Rey Juan Carlos, where an experienced clinical neuropsychologist performed individualized assessments of the study variables (sociodemographic data, psychological data, and neurocognitive tests) for each participant.

### 2.2. Variables and Instruments

*Pain Intensity*. This was measured with an 11-point numerical pain rating scale (NPRS) [22] containing 4 items ranging from 0 (no pain) to 10 (the worst pain you can imagine), where the subjects were asked to indicate the least, the worst, and the average pain intensity experienced in the last week, as well as their current pain.

*Pain-related disability.* This was assessed using the Spanish version of the Fibromyalgia Impact Questionnaire (FIQ) [23]. The FIQ consists of 10 subscales where physical function, number of days not feeling well, missed work, social skills, fatigue, morning tightness, stiffness, anxiety, and depression are evaluated. The total score ranges from 0 to 100 points, where the higher the score, the greater the negative impact of FMS [24]. Only the group of patients with FMS completed this questionnaire.

*Anxiety and depressive symptoms.* The Spanish version of the Hospital Anxiety and Depression Scale (HADS) was used to assess the presence of anxiety and depressive symptoms [25,26]. It consists of two subscales (HADS-A: anxiety; HADS-D: depression) with 7 items each and scores ranging from 0 to 3 [27]. We applied a cut-off score of ≥8 points of each scale because it showed good sensitivity and specificity for determining the presence of anxiety or depressive symptoms [28]. The psychometric properties of both scales for assessing anxiety and depressive symptoms are good [29].

*Sleep quality*. The Spanish version of the Pittsburgh Sleep Quality Index (PSQI) was used to assess sleep quality [30]. The PSQI provides an overall score on sleep quality (from 0 to 21 points) based on 19 questions evaluating different aspects of sleep, such as usual bedtime, wake-up time, number of hours slept, and time needed to fall asleep [31]. A total score ≥8 points indicated that the individual had poor sleep quality.

*Pain hypervigilance.* The Spanish version of the short-form 9-item Pain Vigilance and Awareness Questionnaire (PVAQ-9, score 0 to 45 points) was used to evaluate pain hypervigilance [32].

*Selective attention.* The Spanish version of the D2 Attention test [33] was used to measure selective attention and mind concentration, defined as “the capacity to selectively focus on certain relevant aspects in a task while ignoring other irrelevant ones as well as doing so quickly and accurately” [34]. The D2 test is made up of 14 lines with 47 characters each for a total of 658 items. It contains the letters “d” and “p”, which might appear with one or two little dashes above or below each letter. The subject must carefully check, from left to right, the contents of each line, marking every letter “d” with two little dashes (both above, below, or one above/one below). These are the relevant elements, whereas the remaining combinations (the “p” with or without dashes and the “d” with one or no dash) are considered irrelevant. The subject is given 20 s for each line, and the test usually lasts between 8 and 10 min.

*Visuospatial memory.* The Rey–Osterrieth Complex Figure (ROCF) was used to evaluate visual perception, video-constructional ability, and spontaneous memory retention [35]. The ROCF can evaluate the ability to retain the visual details of the figure, organize and integrate the different parts of the figure, and mentally manipulate the figure [36]. First, participants are requested to copy a geometric figure (18 units of black lines) on a sheet of paper. Then, the sheets are taken away and the participants are asked to draw the figure from memory immediately afterwards (immediate recall) and after 20–30 min (delayed recall). No instructions are provided to memorize the figure because the task intends to measure what is spontaneously kept in mind.

*Working memory.* Working memory was evaluated with the subtest “Digits D/R/I” of the Wechsler Adult Intelligence Scale WAIS-IV battery [37]. This test primarily measures immediate and working memory by assessing sequencing, planning, alertness, and cognitive flexibility skills. The test consists of the following three tasks: digit span forward (DSF, consists of repeating a series of digits, which are presented orally, in the same order they are presented); digit span backward (DSB, repeating a series of digits in the reverse order to those that are presented); and digit span sequencing (DSS, repeating numbers read by the examiner in the lowest to highest order).

*Mental inhibition.* Mental inhibition was evaluated through the “response inhibition index” of the 5-Digit test or FDT [38], a STROOP-type task. The FDT consists of four parts: reading, counting, election, and alternation, which differ in the level of difficulty in the evaluation of executive functions and are applied sequentially. Each part of the test comprises 50 items. The reading and counting parts measure automatic and simple processes, and the alternation and election parts measure more complex processes because they require active mental control and force the individual to expend voluntary effort that reduces the speed of responses.

*Processing speed.* Processing speed was assessed using the subtest “Symbol search” of the WAIS-IV battery [39]. The Symbol Search subtest (SS) of the Wechsler Adult Intelligence Scale is a sensitive test for detecting brain dysfunction. The SS is a paper-and-pencil test. The test sheet consists of two areas: a key area where nine nonsense pairs of digits and symbols are printed and a response area where the digits are randomly ordered with blanks printed. Subjects must fill in the blanks with symbols according to the key as quickly as possible for 120 s.

*Planning/decision making.* Planning/decision making was calculated by using the Zoo Map Test of the BADS battery [40]. The Zoo Map Test measures executive functions and specifically assesses organizational, planning, and problem-solving skills to achieve a goal. The scoring method was designed so that a scoring profile could be calculated for each test with a value range of 0 to 16 (Zoo Map Test). For each version of the test, the number of errors made is subtracted from the sequence score on the test sheet. These scores are combined to provide an overall sequence–error score that does not exceed 16 points. A score between 11 and 16 is considered normal, whereas a score ≤10 indicates some degree of deficiency.

### 2.3. Statistical Analyses

Statistical analyses were performed using SPSS 27 Statistical Software, and results were considered significant at the level *p* < 0.05. The normality assumption was tested using the Kolmogorov–Smirnov test (all data showed a normal distribution). Next, descriptive and frequency analyses were performed for sociodemographic and cognitive variables.

Subsequently, before the execution of the main analyses, a series of preliminary analyses were performed. Firstly, Pearson’s correlation analyses were conducted among sociodemographic and psychological data, as well as neurocognitive tests, with the aim of identifying potential covariates. Anxiety, depression, and sleep quality showed significant associations with the neurocognitive tests (see Section 3) and were therefore included as covariates. Secondly, chi-square analysis for categorial variables and independent samples *t*-test for continuous variables were performed to determine potential intergroup differences in sociodemographic and psychological data.

Then, for the main analyses of this study, a multivariate analysis of covariance (MANCOVA) was conducted to assess the effect of the group on the neurocognitive tests. The variable group was introduced as an independent variable, all neurocognitive tests were considered dependent variables, and anxiety, depression, and sleep quality were introduced as covariates. Subsequently, to evaluate the effect of anxiety, depression, and sleep quality on neurocognitive test indices, three separate multivariate analyses of variance (MANOVAs) were performed. To fulfil this purpose, prior to running the MANOVA analyses, we stratified the variables anxiety, depression, and sleep quality based on the cut-off point for each scale. Next, for the execution of the three MANOVA analyses, the variables group, anxiety, depression, and sleep quality were introduced as independent variables, and the neurocognitive test indices were introduced as dependent variables. Effect sizes were calculated with partial eta squared (n^2^*p*), according to Cohen [20], where a value of 0.01 represents a small effect, a value of 0.06 represents a medium effect, and values greater than 0.14 represent a large effect. A Bonferroni post hoc test was conducted to determine specific intergroup differences.

## 3. Results

### 3.1. Preliminary Analyses

#### 3.1.1. Between-Group Comparisons of Anxiety, Depression, Sleep Quality, and Pain Hypervigilance

A between-group comparison of the psychological variables is shown in Table 1. The results showed significant between-group differences for anxiety (*p* < 0.001, d = 3.987), depressive symptoms (*p* < 0.001, d = 4.009), pain hypervigilance (*p* < 0.001, d = 10.267), and sleep quality (*p* < 0.001, d= 3.933). In addition, significant between-group differences in pain intensity were also found (*p* < 0.001, d = 1.643) The group of women with FMS exhibited higher anxiety levels (mean difference: 7.6, 95%CI 6.6–8.6), higher depressive symptoms (mean difference: 7.5, 95% CI 6.5–8.5), more pain hypervigilance (mean difference: 13.05, 95%CI 10.4–15.7), poor sleep quality (mean difference: 8.0, 95%CI 7.0–9.0), and a higher intensity of pain (mean difference: 6.37, 95%CI 5.9–6.8) than the group of pain-free women.

#### 3.1.2. Pearson’s Correlation Analyses

The correlational analysis revealed that all neurocognitive tests showed significant correlations with anxiety (−0.276 < r < 0.270, all *p* < 0.001), depressive symptoms (−0.260 < r < 0.234, all *p* < 0.001), sleep quality (−0.222 < r < 0.314, all *p* < 0.001), and pain hypervigilance (−0.246 < r < 0.185, all *p* < 0.01).

### 3.2. Main Analyses

#### 3.2.1. Effect of Group on Neurocognitive Tests

The MANCOVA revealed a significant main group effect on neurocognitive tests (Wilk’s λ = 0.874, F_[19,217]_ = 1.645, *p* = 0.048, n^2^_p_ = 0.126, β-1 = 0.942) after controlling for anxiety (Wilk’s λ = 0.919, F_[19,217]_ = 1.006, *p* = 0.455, n^2^_p_ = 0.081, β-1 = 0.723), depression (Wilk’s λ = 0.952, F_[19,217]_ = 0.578, *p* = 0.919, n^2^_p_ = 0.048, β-1 = 0.424), and sleep quality (Wilk’s λ = 0.912, F_[19,217]_ = 1.108, *p* = 0.344, n^2^_p_ = 0.088, β-1 = 0.777).

Univariate analyses with the estimated marginal mean and standard deviations for each neuropsychological test are shown in Table 2. Pairwise comparisons showed that the group of patients with FMS showed lower scores in D2_TR (mean difference: −42.4, SD: 17.9, 95% CI −77.7 to −7.1, *p* = 0.019), D2_TA (mean difference: −21.5, SD: 7.9, 95%CI −37.1 to −5.9, *p* = 0.007), D2_TOT (mean difference: −50.1, SD: 17.7, 95%CI −85.1 to −15.1, *p* = 0.005), D2_CON (mean difference: −25.3, SD: 8.7, 95%CI −42.4 to −8.2, *p* = 0.004), ROCF_Copy (mean difference: −2.3, SD: 1.1, 95%CI −4.5 to −.07, *p* = 0.043), ROCF_Recall (mean difference: −4.0, SD: 1.4, 95%CI −6.7 to −1.3, *p* = 0.004), and Symbol Search (mean difference: −4.5, SD: 1.7, 95%CI −7.8 to −1.2, *p* = 0.008) as compared to the group of pain-free subjects.

In addition, patients with FMS achieved greater significant scores than pain-free subjects in D2_C (mean difference: 4.7, SD: 2.3, 95%CI 0.2 to 9.2, *p* = 0.042), Decoding _FDT (mean difference: 5.1, SD: 1.5, 95%CI 2.2 to 8.1, *p* = 0.001), Retrieving_FDT (mean difference: 7.8, SD: 2.4, 95%CI 3.0 to 12.5, *p* = 0.001), or Inhibiting_FDT (mean difference: 8.9, SD: 3.9, 95%CI 1.2 to 16.6, *p* = 0.024).

#### 3.2.2. Anxiety and Group Effects on Neurocognitive Tests

The MANOVA analyses found a significant main group effect (Wilk’s λ = 0.855, F_[19,219]_ = 1.947, *p* = 0.012, n^2^*p* = 0.145, β-1 = 0.976) but not a main effect of anxiety (Wilk’s λ = 0.911, F_[19,219]_ = 1.120, *p* = 0.332, n^2^*p* = 0.089, β-1 = 0.783) nor an interaction effect between group and anxiety (Wilk’s λ = 0.883, F_[19,219]_ = 1.530, *p* = 0.077, n^2^*p* = 0.117, β-1 = 0.92) on the neurocognitive tests (Table 3).

#### 3.2.3. Depression and Group Effects on Neurocognitive Tests

The MANOVA analyses revealed a significant main group effect (Wilk’s λ = 0.859, F_[19,219]_ = 1.896, *p* = 0.016, n^2^*p* = 0.141, β-1 = 0.972), but neither a main effect of depressive symptoms (Wilk’s λ = 0.936, F_[19,219]_ = 0.792, *p* = 0.715, n^2^*p* = 0.064, β-1 = 0.587) nor a group and depression interaction (Wilk’s λ = 0.948, F_[19,219]_ = 0.627, *p* = 0.884, n^2^*p* = 0.052, β-1 = 0.463) on neurocognitive tests (Table 4).

#### 3.2.4. Sleep Quality and Group Effects on Neurocognitive Tests

The MANOVA found a significant group effect (Wilk’s λ = 0.853, F_[19,219]_ = 1.982, *p* = 0.010, n^2^*p* = 0.147, β-1 = 0.979), but not a main effect of sleep quality (Wilk’s λ = 0.911, F_[19,219]_ = 1.131, *p* = 0.321, n^2^*p* = 0.089, β-1 = 0.788) nor a group and sleep quality interaction effect (Wilk’s λ = 0.921, F_[19,219]_ = 0.986, *p* = 0.478, n2p = 0.079, β-1 = 0.713) on the neurocognitive tests. Table 5 shows the estimated marginal mean and standard deviations according to the presence or absence of poor sleep in both groups.

## 4. Discussion

This research found that the group of patients with FMS exhibited worse cognitive performance in comparison to the group of pain-free subjects. Furthermore, it was also observed that differences were minimally affected by anxiety, depression, and sleep quality. The presence of cognitive impairments at different domains in women with FMS is supported by the previous literature [8,9]. In this regard, significant differences were observed in selective attention, long-term visual memory, processing speed, and mental inhibition but not in the executive functions of working memory and planning.

Thus, our results found that women with FMS exhibit worse processing speed, a less amount of work, and less personal motivation, as expressed by lower D2 attention test scores, than pain-free women. Similarly, the indices related to the amount of work and precision of processing, inhibitory attentional control, cognitive flexibility, concentration, and balance between mental speed and precision were significantly different between women with and without FMS. Our results agree with those of previous studies showing that patients with FMS had worse scores in attention, processing speed, long-term memory, and mental inhibition, but our findings disagree with differences in working memory [41,42]. Thus, it should be noted that the most notable difference between women with and without FMS at the executive level was seen in difficulties at the inhibitory level, in agreement with the review by Mendonça et al. [6], where almost 100 executive functions were analyzed in patients with FMS, and the most significant result identified a deficit regarding inhibitory control. The aforementioned results agree with previous meta-analyses showing that cognitive problems in patients with FMS do not affect all domains and executive functions at the same level [8,9]. González-Villar et al. [41] found that patients with FMS exhibit lower functional connectivity between midfrontal locations and the rest of the scalp-recorded areas in the theta band, areas related to information transfer across distant brain regions when top–down control (inhibition) is required. Thus, patients with FMS also exhibit abnormalities in their frontoparietal networks; these brain changes may explain some of the cognitive impairments identified in women with FMS [43].

Previous and current data suggest that patients with FMS have a generalized worse quality of attention as well as difficulties with the speed of information processing, following instructions, memory, inhibition capacity, and execution of complex tasks related to visual discrimination of stimuli, which may be part of “fibrofog”. Fibrofog refers to the symptomatology that brings together a set of cognitive complaints (e.g., memory problems, attention deficits, issues with orientation, and general confusion) self-reported by patients with FMS [44,45]. Nevertheless, it should be noted that fibrofog can affect different cognitive aspects in each patient, and this would agree with the results of this current study.

In addition, cognitive disturbances and executive functions can also be mediated or aggravated by the presence of psychological disturbances such as anxiety, depression, fatigue, or poor-quality sleep. An important finding of this study was that cognitive differences between women with and without FMS were not affected by anxiety, depression, or poor sleep. The association between mood disorders and cognitive impairments in patients with FMS is conflicting. Studies conducted by the same research group reported that cognitive impairments are related to anxiety and depression levels [10,11]. However, these authors recognized that the percentage of explanations was small and that differences between people with and without FMS were still significant (particularly for subjective variables), independent of anxiety and depressive levels [10]. By contrast, Dick et al. [12] did not find an effect of anxiety or depression on cognitive performance in their cohort of patients with FMS. Nevertheless, the presence of anxiety and depressive symptoms in women with FMS is supported in the literature [46]. In fact, most women in the FMS group in our study showed anxiety (89%) and depressive (77.7%) symptoms. Accordingly, the fact that most women with FMS exhibited mood disorders could explain why no significant effects were identified because the group of women with FMS without mood disorders was small. Another potential explanation is that previous studies used different neurocognitive tests and different self-reported questionnaires for assessing anxiety/depressive symptoms than those used in our study.

The role of sleep quality in cognitive impairments and executive functions, albeit less investigated than mood disorders, also reveals heterogeneous results. Grace et al. [15] and Dick et al. [12] did not find an effect of sleep quality on cognitive performance. By contrast, Miró et al. [16] observed that sleep dysfunction was a predictor of alertness but not of vigilance. Again, almost 90% of our sample of women with FMS reported poor sleep quality. Accordingly, heterogeneity in the variables, the use of subjective against objective assessments, different anxiety/depressive levels or sleep quality among samples, and differences in FMS diagnostic criteria could explain these discrepancies.

The results of this study should be considered in terms of potential limitations. First, multiple comparisons were performed, and this strategy increased the likelihood of Type I error. However, given the observational nature of this current study, we applied restricted multivariate analyses. Second, we only included women with FMS to provide greater homogeneity in the sample; however, studies in men with FMS should be conducted. Third, the reduced number of women with FMS without anxiety levels, depressive symptoms, or poor sleep is another limitation that could explain the lack of associations. Fourth, we cannot exclude the long-lasting effect of medications that patients have taken in the past on cognitive performance, although we excluded patients actively taking psychoactive drugs or other medications that may directly affect cognitive function. Finally, we included a battery of neurocognitive tests that could be not sensitive to specific cognitive impairment in women with FMS. In fact, it has been previously suggested that unexpected results may be explained by underpowered studies or by looking at the wrong targets. Therefore, further studies need to be conducted using homogeneous patient samples and a wider battery of neuropsychological tests capable of covering all the components of executive functions and attentional and memory processes.

## 5. Conclusions

This study found that patients with FMS exhibited worse cognitive performance and executive function particularly in the cognitive domains of selective attention, long-term visual memory, processing speed, and mental inhibition in comparison to the group of pain-free women. Thus, cognitive performance did not seem to be related to anxiety, depression, or sleep quality in our sample of women with FMS.

## Figures and Tables

**Table 1:** Demographic and psychological data of the sample.

	Fibromyalgia Syndrome (n = 130)	Pain-Free Subjects (n = 111)	χ^2^	*p*
Marital Status			0.043	0.979
Single	25 (19.20%)	21 (18.90%)		
Married	92 (70.80%)	78 (70.30%)		
Widowed	13 (10%)	12 (10.80%)		
Educational Level			0.052	0.974
Primary	34 (26.20%)	30 (27%)		
Secondary	51 (39.20%)	42 (37.80%)		
Higher Education	45 (34.60%)	39 (35.10%)		
Race			0.401	0.527
Caucasian	126 (96.90%)	109 (98.20%)		
Latin American	4 (3.10%)	2 (1.80%)		
Employment Status			42.349	0.000
Working	40 (30.80%)	65 (58.60%)		
Housewives	11 (8.50%)	10 (9%)		
Unemployed	36 (27.70%)	8 (7.20%)		
Medical leave	26 (20%)	3 (2.70%)		
Retired	17 (13.10%)	25 (22.50%)		
	Mean (SD)	Mean (SD)	t	*p*
Age	54.67 (9.430)	55.31 (13.969)	−0.408	0.684
NPRS (0–10)	7.61 (1.309)	1.23 (1.964)	29.122	0.000
HADS-A (0–21)	13.52 (3.916)	5.93 (4.069)	14.724	0.000
HASD-D (0–21)	11.05 (4.304)	3.52 (3.633)	14.716	0.000
PVAQ-9 (0–45)	29.40 (7.791)	16.35 (12.564)	9.494	0.000
PSQI (0–21)	15.19 (3.775)	7.23 (4.110)	15.659	0.000
FIQ (0–100)	75.18 (12.207)	-----		

Note: SD = Standard Deviation; NPRS: Numerical Pain Rating Scale; HADS: Hospital Anxiety and Depression Scale (A: anxiety, D: depression); PVAQ-9: Pain Vigilance and Awareness Questionnaire; PSQI: Pittsburg Sleep Quality Index; FIQ = Fibromyalgia Impact Questionnaire; χ^2^: Chi-Square Statistic; t: Independent Samples *t*-test.

**Table 2:** Estimated marginal means and standard deviations of neurocognitive tests.

Neurocognitive Indices	Fibromyalgia Syndrome (n = 130) Mean (SD)	Pain-Free Subjects (n = 111) Mean (SD)	F	*p*	n^2^*p*	β-1
d2_TR	337.125 (10.163)	379.529 (11.341)	5.596	0.019	0.023	0.654
d2_TA	110.169 (4.487)	131.685 (5.007)	7.390	0.007	0.030	0.773
d2_O	31.955 (3.768)	29.476 (4.204)	0.139	0.709	0.001	0.066
d2_C	6.555 (1.310)	1.828 (1.462)	4.184	0.042	0.017	0.531
d2_TOT	295.579 (10.072)	345.646 (11.239)	7.943	0.005	0.033	0.801
d2_CON	104.907 (4.930)	130.236 (5.502)	8.484	0.004	0.035	0.827
d2_VAR	15.642 (0.796)	14.662 (0.888)	0.487	0.486	0.002	0.107
DSF	7.893 (0.219)	7.981 (0.245)	0.051	0.821	0.000	0.056
DSB	6.758 (0.211)	7.302 (0.235)	2.135	0.145	0.009	0.307
DSS	7.160 (0.241)	7.479 (0.269)	0.561	0.455	0.002	0.116
ROCF_Copy	30.457 (0.642)	32.762 (0.717)	4.137	0.043	0.017	0.526
ROCF_Recall	11.988 (0.778)	15.987 (0.869)	8.485	0.004	0.035	0.827
ROCF_TimeCopy	3.135 (1.113)	3.913 (1.242)	0.157	0.692	0.001	0.068
Symbol Search	26.437 (0.953)	30.939 (1.063)	7.181	0.008	0.030	0.761
Decoding_FDT	25.308 (0.836)	20.153 (0.933)	12.224	0.001	0.049	0.936
Retrieving_FDT	29.765 (1.372)	21.987 (1.531)	10.331	0.001	0.042	0.893
Inhibiting_FDT	46.869 (2.224)	37.947 (2.482)	5.172	0.024	0.022	0.620
Shifting_FDT	60.652 (2.973)	50.462 (3.317)	3.777	0.053	0.016	0.490
Zoo Map test	11.061 (0.426)	12.090 (0.476)	1.874	0.172	0.008	0.276

Note: SD = Standard Deviation; d2_TR = total number of items answered; d2_TA = number of items answered correctly; d2_O = errors of omission committed; d2_C = commission errors made; d2_TOT = number of elements processed minus the total number of errors committed; d2_CON = number of relevant elements marked minus the number of commissions; d2_VAR = variation index d2; DSF = Digit Span Forward; DSB = Digit Span Backward; DSS = Digit Span Sequencing; ROCF_Copy = direct scoring in the copy phase of the Rey–Osterrieth Complex Figure; ROCF_Recall = direct scoring in the delayed Recall phase of the Rey–Osterrieth Complex Figure; Symbol Search = direct scoring of correctly answered items; Decoding_FDT = time in seconds to read all numeric items; Retrieving_FDT = time in seconds to read all non-numeric items; Inhibiting_FDT = time in seconds to read numeric items; Shifting_FDT = time in seconds to read non-numeric items; Zoo Map Test = direct score in carrying out the planning test.

**Table 3:** Joint effects of anxiety and group on the results of the neurocognitive tests.

Neurocognitive Indices	Fibromyalgia Syndrome (n = 130)				Pain-Free Subjects (n = 111)			
	No Significant Anxiety (n = 14) Mean (SD)	Significant Anxiety (n = 116) Mean (SD)	*p*	n^2^*p*	No Significant Anxiety (n = 77) Mean (SD)	Significant Anxiety (n = 34) Mean (SD)	*p*	n^2^*p*
d2_TR	342.500 (24.485)	332.371 (8.506)	0.696	0.001	379.935 (10.441)	392.618 (15.712)	0.502	0.002
d2_TA	117.000 (10.870)	106.819 (3.776)	0.377	0.003	130.870 (4.635)	142.147 (6.975)	0.179	0.008
d2_O	27.214 (9.150)	33.759 (3.179)	0.500	0.002	30.013 (3.901)	24.059 (5.871)	0.399	0.003
d2_C	7.071 (3.189)	4.586 (1.108)	0.462	0.002	4.701 (1.360)	1.824 (2.046)	0.243	0.006
d2_TOT	307.071 (24.326)	289.724 (8.451)	0.501	0.002	346.649 (10.373)	358.618 (15.610)	0.524	0.002
d2_CON	112.357 (11.921)	103.069 (4.141)	0.462	0.002	126.558 (5.083)	141.765 (7.650)	0.099	0.011
d2_VAR	14.241 (1.940)	15.647 (0.674)	0.486	0.002	14.961 (0.827)	14.559 (1.245)	0.788	0.000
DSF	7.571 (0.536)	7.603 (0.186)	0.955	0.000	8.364 (0.229)	8.235 (0.344)	0.756	0.000
DSB	6.357 (0.512)	6.655 (0.178)	0.583	0.001	7.545 (0.218)	7.265 (0.329)	0.478	0.002
DSS	6.286 (0.584)	6.862 (0.203)	0.352	0.004	8.221 (0.249)	7.176 (0.375)	0.021	0.022
ROCF_Copy	29.857 (1.556)	31.069 (0.544)	0.466	0.002	32.117 (0.668)	32.382 (1.005)	0.826	0.000
ROCF_Recall	10.643 (1.884)	12.759 (0.665)	0.290	0.005	15.117 (0.804)	15.882 (1.209)	0.599	0.001
ROCF_TimeCopy	3.269 (2.693)	3.090 (0.936)	0.950	0.000	4.534 (1.148)	2.602 (1.728)	0.353	0.004
Symbol Search	28.286 (2.295)	26.112 (0.797)	0.372	0.003	30.974 (0.978)	31.206 (1.472)	0.896	0.000
Decoding_FDT	25.071 (2.029)	25.621 (0.705)	0.798	0.000	19.805 (0.865)	19.971 (1.302)	0.916	0.000
Retrieving_FDT	35.857 (3.311)	28.741 (1.150)	0.043	0.017	22.091 (1.412)	22.735 (2.125)	0.801	0.000
Inhibiting_FDT	52.857 (5.371)	47.707 (1.866)	0.366	0.003	35.130 (2.290)	39.000 (3.446)	0.351	0.004
Shifting_FDT	69.357 (7.173)	61.026 (2.492)	0.274	0.005	47.896 (3.059)	51.412 (4.603)	0.525	0.002
Zoo Map test	10.571 (1.023)	10.991 (0.355)	0.698	0.001	12.636 (0.436)	11.294 (0.656)	0.090	0.012

Note: SD = Standard Deviation; d2_TR = total number of items answered; d2_TA = number of items answered correctly; d2_O = errors of omission committed; d2_C = commission errors made; d2_TOT = number of elements processed minus the total number of errors committed; d2_CON = number of relevant elements marked minus the number of commissions; d2_VAR = variation index d2; DSF = Digit Span Forward; DSB = Digit Span Backward; DSS = Digit Span Sequencing; ROCF_Copy = direct scoring in the copy phase of the Rey–Osterrieth Complex Figure; ROCF_Recall = direct scoring in the delayed Recall phase of the Rey–Osterrieth Complex Figure; Symbol Search = direct scoring of correctly answered items; Decoding_FDT = time in seconds to read all numeric items; Retrieving_FDT = time in seconds to read all non-numeric items; Inhibiting_FDT = time in seconds to read numeric items; Shifting_FDT = time in seconds to read non-numeric items; Zoo Map Test = direct score in carrying out the planning test.

**Table 4:** Joint effects of depression and group on the results of the neurocognitive tests.

Neurocognitive Indices	Fibromyalgia Syndrome (n = 130)				Pain-Free Subjects (n = 111)			
	No Significant Depression (n = 29) Mean (SD)	Significant Depression (n = 101) Mean (SD)	*p*	n^2^*p*	No Significant Depression (n = 95) Mean (SD)	Significant Depression (n = 16) Mean (SD)	*p*	n^2^*p*
d2_TR	317.655 (16,984)	338.000 (9.101)	0.292	0.005	381.863 (9.384)	395.437 (22.865)	0.583	0.001
d2_TA	110.724 (7.591)	107.109 (4.068)	0.675	0.001	134.147 (4.194)	135.375 (10.220)	0.912	0.000
d2_O	23.207 (6.327)	35.881 (3.390)	0.079	0.013	27.400 (3.496)	32.875 (8.518)	0.553	0.001
d2_C	5.793 (2.221)	4.584 (1.190)	0.632	0.001	4.137 (1.227)	1.937 (2.991)	0.497	0.002
d2_TOT	289.207 (16.915)	292.277 (9.064)	0.873	0.000	347.884 (9.346)	364.750 (22.772)	0.494	0.002
d2_CON	106.414 (8.337)	103.396 (4.467)	0.750	0.000	130.842 (4.606)	133.437 (11.224)	0.831	0.000
d2_VAR	14.828 (1.347)	15.683 (0.722)	0.576	0.001	14.621 (0.744)	16.125 (1.813)	0.444	0.002
DSF	7.517 (0.372)	7.624 (0.199)	0.801	0.000	8.368 (0.206)	8.062 (0.501)	0.573	0.001
DSB	6.724 (0.356)	6.594 (0.191)	0.748	0.000	7.432 (0.197)	7.625 (0.480)	0.748	0.000
DSS	6.448 (0.409)	6.901 (0.219)	0.331	0.004	8.000 (0.226)	7.312 (0.551)	0.250	0.006
ROCF_Copy	27.862 (1.066)	31.822 (0.571)	0.001	0.043	32.147 (0.589)	32.500 (1.435)	0.820	0.000
ROCF_Recall	11.966 (1.310)	12.693 (0.702)	0.625	0.001	15.105 (0.724)	16.812 (1.764)	0.372	0.003
ROCF_TimeCopy	3.260 (1.873)	3.066 (1.004)	0.927	0.000	4.186 (1.035)	2.496 (2.522)	0.536	0.002
Symbol Search	24.793 (1.593)	26.792 (0.853)	0.270	0.005	30.947 (0.880)	31.625 (2.144)	0.770	0.000
Decoding_FDT	26.586 (1.408)	25.267 (0.754)	0.410	0.003	19.832 (0.778)	20.000 (1.895)	0.935	0.000
Retrieving_FDT	32.414 (2.310)	28.673 (1.238)	0.155	0.009	22.105 (1.276)	23.375 (3.110)	0.706	0.001
Inhibiting_FDT	52.448 (3.729)	47.059 (1.998)	0.204	0.007	35.789 (2.060)	39.437 (5.020)	0.502	0.002
Shifting_FDT	67.172 (4.986)	60.416 (2.672)	0.233	0.006	48.905 (2.755)	49.375 (6.712)	0.948	0.000
Zoo Map test	10.931 (0.715)	10.950 (0.383)	0.981	0.000	12.221 (0.395)	12.250 (0.963)	0.978	0.000

Note: SD = Standard Deviation; d2_TR = total number of items answered; d2_TA = number of items answered correctly; d2_O = errors of omission committed; d2_C = commission errors made; d2_TOT = number of elements processed minus the total number of errors committed; d2_CON = number of relevant elements marked minus the number of commissions; d2_VAR = variation index d2; DSF = Digit Span Forward; DSB = Digit Span Backward; DSS = Digit Span Sequencing; ROCF_Copy = direct scoring in the copy phase of the Rey–Osterrieth Complex Figure; ROCF_Recall = direct scoring in the delayed Recall phase of the Rey–Osterrieth Complex Figure; Symbol Search = direct scoring of correctly answered items; Decoding_FDT = time in seconds to read all numeric items; Retrieving_FDT = time in seconds to read all non-numeric items; Inhibiting_FDT = time in seconds to read numeric items; Shifting_FDT = time in seconds to read non-numeric items; Zoo Map Test = direct score in carrying out the planning test.

**Table 5:** Joint effects of sleep quality and group on the results of the neurocognitive tests.

Neurocognitive Indices	Fibromyalgia Syndrome (n = 130)				Pain-Free Subjects (n = 111)			
	No Poor Sleep Quality (n = 15) Mean (SD)	Poor Sleep Quality (n = 115) Mean (SD)	*p*	n^2^*p*	No Poor Sleep Quality (n = 71) Mean (SD)	Poor Sleep Quality (n = 40) Mean (SD)	*p*	n^2^*p*
d2_TR	321.200 (23.655)	335.061 (8.543)	0.582	0.001	380.197 (10.873)	390.250 (14.486)	0.579	0.001
d2_TA	93.600 (10.485)	109.783 (3.787)	0.148	0.009	131.099 (4.819)	140.050 (6.421)	0.266	0.005
d2_O	36.600 (8.849)	32.591 (3.196)	0.670	0.001	29.831 (4.067)	25.275 (5.419)	0.502	0.002
d2_C	10.267 (3.070)	4.148 (1.109)	0.062	0.015	3.620 (1.411)	4.175 (1.880)	0.813	0.000
d2_TOT	267.933 (23.446)	294.678 (8.468)	0.284	0.005	344.423 (10.777)	360.775 (14.358)	0.363	0.003
d2_CON	86.533 (11.506)	106.357 (4.156)	0.106	0.011	127.901 (5.289)	137.100 (7.046)	0.298	0.005
d2_VAR	18.067 (1.867)	15.157 (0.674)	0.144	0.009	15.014 (0.858)	14.525 (1.143)	0.733	0.000
DSF	8.333 (0.515)	7.504 (0.186)	0.131	0.010	8.437 (0.237)	8.125 (0.315)	0.430	0.003
DSB	7.533 (0.490)	6.504 (0.177)	0.050	0.016	7.296 (0.225)	7.750 (0.300)	0.228	0.006
DSS	7.000 (0.571)	6.774 (0.206)	0.710	0.001	8.028 (0.262)	7.675 (0.350)	0.420	0.003
ROCF_Copy	29.733 (1.512)	31.096 (0.546)	0.398	0.003	32.056 (0.695)	32.540 (0.926)	0.734	0.000
ROCF_Recall	11.733 (1.825)	12.635 (0.659)	0.643	0.001	15.211 (0.839)	15.600 (1.117)	0.781	0.000
ROCF_TimeCopy	3.244 (2.588)	3.092 (0.935)	0.956	0.000	2.618 (1.189)	6.292 (1.585)	0.065	0.014
Symbol Search	24.667 (2.216)	26.565 (0.800)	0.421	0.003	30.746 (1.019)	31.575 (1.357)	0.626	0.001
Decoding_FDT	23.533 (1.955)	25.826 (0.706)	0.271	0.005	19.831 (0.899)	19.900 (1.197)	0.963	0.000
Retrieving_FDT	25.133 (3.212)	30.078 (1.160)	0.149	0.009	21.944 (1.476)	22.900 (1.967)	0.698	0.001
Inhibiting_FDT	44.467 (5.199)	48.757 (1.878)	0.439	0.003	35.817 (2.390)	37.200 (3.184)	0.729	0.001
Shifting_FDT	55.667 (6.939)	62.739 (2.506)	0.339	0.004	48.408 (3.189)	49.975 (4.249)	0.768	0.000
Zoo Map test	11.267 (0.994)	10.904 (0.359)	0.732	0.000	12.338 (0.457)	12.025 (0.609)	0.681	0.001

Note: SD = Standard Deviation; d2_TR = total number of items answered; d2_TA = number of items answered correctly; d2_O = errors of omission committed; d2_C = commission errors made; d2_TOT = number of elements processed minus the total number of errors committed; d2_CON = number of relevant elements marked minus the number of commissions; d2_VAR = variation index d2; DSF = Digit Span Forward; DSB = Digit Span Backward; DSS = Digit Span Sequencing; ROCF_Copy = direct scoring in the copy phase of the Rey–Osterrieth Complex Figure; ROCF_Recall = direct scoring in the delayed Recall phase of the Rey–Osterrieth Complex Figure; Symbol Search = direct scoring of correctly answered items; Decoding_FDT = time in seconds to read all numeric items; Retrieving_FDT = time in seconds to read all non-numeric items; Inhibiting_FDT = time in seconds to read numeric items; Shifting_FDT = time in seconds to read non-numeric items; Zoo Map Test = direct score in carrying out the planning test.

## Data Availability

The materials and analysis code for this study are not available in any repository; however, we will make our data accessible upon request to the corresponding author.
